# Neighbourhood greenness moderates the association between physical activity and geriatric-relevant health outcomes: an analysis of the CLSA

**DOI:** 10.1186/s12877-023-03997-w

**Published:** 2023-05-22

**Authors:** Andrew Putman, Irmina Klicnik, Shilpa Dogra

**Affiliations:** University of Ontario Institute of Technology, 2000 Simcoe St N, Oshawa, ON L1G 0C5 Canada

**Keywords:** Pain, Polypharmacy, Depression, Physical function, CLSA

## Abstract

**Background:**

The purpose of this analysis was to evaluate the relationship between baseline physical activity levels of older adults and geriatric-relevant health outcomes at 3-year follow-up, and to determine whether baseline neighbourhood characteristics alter this association.

**Methods:**

Data from the Canadian Longitudinal Study on Aging (CLSA) were used to assess geriatric-relevant outcomes of physical impairment, medication use, severity of daily pain, and depressive symptoms. Data from the Canadian Active Living Environments (Can-ALE) and the Normalized Difference Vegetative Index (NDVI) were used to determine neighbourhood walkability and greenness, respectively. The analytic sample included adults who were 65 years or older at baseline $(n=\text{16,735}, age=73\pm 5.6, 50\% female)$. Adjusted odds ratios and 95% confidence intervals for the base relationships were calculated using proportional odds logistic regression (physical impairment, pain, medication use), and linear regression (depressive symptoms). Moderation effects of environmental factors were assessed using greenness and walkability.

**Results:**

The base relationships showed protective associations between each additional hour per week of total physical activity and physical impairment $\left(OR=0.95, 95\%CI=\text{0.94,0.96}; p<.001\right),$ daily pain severity $\left(OR=0.98, 95\%CI=0.98, 0.99; p<.001\right),$ medication use $(OR=0.98, 95\%CI= 0.97, 0.99; p< .001)$, and depressive symptoms $(OR=0.95, 95\%CI=\text{0.94,0.96}; p<.001)$. Additive moderation effects were seen when greenness was added to physical impairment $(\beta =0.022, p=.04)$, daily pain severity $(\beta =0.019, p<.01)$, and depressive symptoms $(\beta =0.032, p=.01)$ but no moderation was seen with walkability. Sex differences were observed. For example, greenness moderation was found in severity of daily pain in males but not in females.

**Conclusion:**

Future research investigating geriatric-relevant health outcomes and physical activity should consider neighbourhood greenness as a potential moderator.

## Introduction

In 2020, the Public Health Agency of Canada reported that there were more than 6 million Canadian adults who were 65 years of age or older [1]. It is anticipated that this number will grow to approximately 11 million, that is, one quarter of Canada’s projected population, by 2040 [1]. Nearly three quarters of Canadian older adults have been diagnosed with one of the ten most prevalent chronic diseases in Canada, and more than one third of older adults have been diagnosed with multiple chronic diseases [1]. Such chronic comorbidity is associated with disability, frailty, and social limitations [2].

It is important to note that diagnosis of a chronic disease alone does not reflect the overall health and wellbeing of an older adult. In fact, some older adults living with multiple chronic diseases report being in excellent health [3]. A study of Dutch older adults (n = 705) found that among those who reported diagnosis with at least one chronic disease, only 227(32%) reported fair or worse self-perceived health, while 478(68%) reported feeling in good or very good health [4]. In order to more accurately describe the extent to which older adults’ health and well-being are being impacted by chronic disease and age-related decline, the conceptual framework of ***geriatric syndromes*** was developed [5]. Geriatric syndromes refer to multifactorial health conditions among older adults that impair their health and well-being but may not fit into a singular disease criterion [6]. It has been suggested that research on older adults shift away from chronic disease outcomes and employ the use of ***geriatric-relevant health outcomes*** to better capture the impacts of exposures and interventions [7].

Research has consistently shown that measures of geriatric-relevant health outcomes are useful in understanding important health outcomes such as the need for assisted living, post-operative care, and overall mortality among older adults [8–10]. Geriatric-relevant health outcomes can be organized into physical function, cognitive function, mental health, social health, and sleep [7]. These outcomes are positively affected by regular physical activity, particularly physical function and mental health [11–13]. However, there are some gaps that need to be addressed. For example, a recent review on pain and physical activity noted that the overall downward trend observed for pain severity with increased physical activity had a high level of variability across individuals, making small and/or under-powered sample sizes a large limitation of the research they assessed [14]. Importantly, despite the high prevalence of chronic pain among older adults, there is a dearth of data available on pain and physical activity on older adults who are living in the community [15]. Another noteworthy gap pertains to medication use, an indicator of the severity or control level of multi-morbidity. Here again, larger studies and more geographical diversity is needed to determine whether these associations are generalizable [16–18].

A growing body of research indicates that geriatric-relevant health outcomes may be influenced by the neighbourhood in which community-dwelling older adults reside. There is some evidence to suggest that neighbourhood greenness is associated with positive mental health and social outcomes, while walkability is associated with better physical function[19, 20]. Similarly, the amount of physical activity community-dwelling older adults engage in is associated with the environmental characteristics of the neighbourhood in which they reside [19, 21, 22]. For example, a more walkable neighbourhood or a neighbourhood with more trees may encourage older adults to walk more [23]. Neighbourhoods that are age-friendly and provide third places, that is, places other than home or work where people gather for social or recreational purposes, have been shown to be associated with significant physical and social health benefits [7, 24]. A gap in our current knowledge, however, is whether the combination of physical activity and neighbourhood characteristics have an additive moderation effect on geriatric-relevant health outcomes.

Understanding these additive moderation effects may be useful in informing the design of age-friendly communities and interventions that target outdoor physical activity aimed at improving geriatric-relevant health outcomes. For example, a recent systematic review showed that gardening, green exercise, and nature-based therapy were effective for mental health outcomes in adults; little is known of whether similar effects are observed for other geriatric-relevant health outcomes [25]. Moreover, a recent analysis by our group showed that neighbourhood characteristics were not associated with self-reported chronic disease, but did influence self-rated measures of healthy aging [23]. Thus, the purpose of this analysis was to assess the associations between physical activity and the geriatric-relevant health outcomes of physical function, pain, depressive symptoms, and medication use in older Canadians, and then to assess whether the environmental factors of greenness and walkability further moderate those relationships. Given the established sex-differences in the effects of physical activity on health, we also conducted separate subgroup analyses for males and females [26].

## Methods

### Data source and participants

The Canadian Longitudinal Study on Aging (CLSA) is a cross-Canada prospective cohort study of 51,338 community dwelling adults aged 45 to 85 years old when baseline measurements were completed in 2015 [27]. The first set of follow-up measurements were completed in 2018 and additional follow-up measurements are to be conducted every 3 years until 2033 or the participant’s death. All CLSA participants are in one of two separate cohorts, the Tracking cohort and/or the Comprehensive cohort. The Tracking cohort consists of 21,141 randomly selected older adults across all 10 provinces, who were interviewed by phone [27]. The Comprehensive cohort consists of 30,097 participants who were randomly selected from the population residing within 25-50 km of one of the 11 data collection sites, spanning 7 of the 10 provinces; they completed a questionnaire by phone as well as an in-home interview and on-site physical assessment [27]. Persons living on First Nations reserves, full-time members of the Canadian Armed Forces, people living in institutions such as long-term care facilities, and people residing in any of the 3 territories were excluded from the study [27]. Individuals were also excluded from the baseline intake if they were experiencing cognitive impairment or were otherwise unable to understand and give full informed consent themselves [28]. Full details of the CLSA’s methodology, protocol, and sampling technique have been previously published [27]. The CLSA data used in this study were from 2015 to 2018 (dataset version 4.1 and 3.0) and have been linked by 3-character forward sortation area with measurements of the Canadian Active Living Environment (Can-ALE) and Normalized Difference Vegetation Index (NDVI) from the Canadian Urban Environmental Health Research Consortium [29].

For the present analysis, we used a subset of the CLSA data. Those included were 65 or older when their initial measurements were recorded (n = 21,491), had follow-up measurements (n = 18,111), and did not move between their baseline measurement and their first follow-up measurement (n = 16,735). Since the missingness of the variables used in analyses were all less than 5%, multiple imputation with chained equations was used to impute any missing values. The resulting analytic sample included 16,735 participants (females n = 8,372; males n = 8, 363; only two options provided for sex, no missing data) [30].

### Geriatric-relevant health outcomes

*Physical Impairment (at follow-up)*: The severity of limitation in activities of daily living that community-dwelling older adults were experiencing was measured using the Older Americans’ Resources and Services Multidimensional Functional Assessment Questionnaire [13, 18, 22]. The participant’s responses from the first follow-up were scored and categorized into 5 categories: no functional impairment, mild impairment, moderate impairment, severe impairment, and total impairment [31]. Participants in the mild impairment category can complete most of their normal activities of daily living, whereas participants in the severe and total impairment categories may not be able to complete any of their normal daily activities independently.

#### Severity of Daily Pain (at follow-up)

The severity of pain that a participant typically feels was measured in the General Symptoms and Signs section of the first follow-up questionnaire, which has been adapted from the World Health Organization’s International Statistical Classification of Diseases and Related Health Problems 10th Edition [32]. Participants were first asked whether they usually are free from pain and discomfort, and if not, the participant was asked to categorize their usual intensity of pain as either mild, moderate, or severe [32].

#### Number of Prescription Medications (at follow-up)

Participants who met our selection criteria and are part of the Tracking cohort (n = 6,256) were asked at the first follow-up if they had taken any prescription medications in the last 30 days [32]. If they had taken any prescription medications, they were asked if they had taken one, two, or three or more different prescription medications [32].

#### Depressive Symptoms (at follow-up)

Current levels of depression were assessed by the 10-question version of the Center for Epidemiologic Studies Short Depression Scale [CES-D10] [33]. Scores from the CES-D10 can range from 0 (no depressive symptoms) to 30 (the most severe depressive symptoms) [33].

### Exposure variables

#### Total Physical Activity (at baseline)

A modified version of the Physical Activity Scale for the Elderly (PASE) was used to ascertain the typical weekly volumes of physical activity [30]. This questionnaire asked about the frequency and duration of physical activity in light, moderate, and high intensity sports, strength training, and walking. To estimate total hours of weekly physical activity we multiplied the midpoints of the frequency and duration for each category, and then summed those results [23]. The only exception to the use of midpoints was if a participant categorized their typical duration of activity as 4 h or longer; it was encoded as 4 h of activity to help reduce overestimation of total physical activity [23].

#### Greenness (at baseline)

The greenness of an area can be assessed using satellite measurement of the amount of green vegetation within a specified distance of residential buildings [22]. The mean of the annual mean NDVI within 500 m of residences that was linked by forward sortation area [the first 3 characters of the postal code] was used for analysis [29, 30]. The scores were divided into quartiles for analysis (0.001–0.337, 0.338–0.414, 0.415–0.493, and 0.494–0.743) with lower quartiles indicating lower density of greenness.

*Walkability (at baseline)*: The Can-ALE index is a composite measure of the walkability of an environment based on the intersection density, dwelling density, and number of points of interest within 1 km^2^ [29]. The composite measures are then ordered into 5 categories: very low, low, moderate, and high/very high (11.0%; categories collapsed in-line with previous use of Can-ALE data with the CLSA) [23].

### Covariates

All analyses were further stratified by self-reported sex to facilitate sex-based analysis. Age, education, and household income levels were used as covariates. Highest level of education attained was reported using 11 categories: grade 8 or less, grade 9–10, grade 11–13, high school diploma, some post-secondary, trade certificate/diploma, college diploma, university non-degree certificate, bachelor’s degree, graduate degree, or other [30]. Annual household income was categorized as less than $20,000, $20,000 to less than $50,000, $50,000 to less than $100,000, $100,000 to less than $150,000, or $150,000 or greater [30].

### Statistical analysis

The statistical computing language *R* version 4.2.0, and the packages *tidyverse* version 1.3.1, *MASS* version 7.3–57, and *mice* version 3.14.0 were used to compute the statistical analyses in this study [34–37]. Descriptive statistics were calculated before imputation, with the categorical variable presented as percentages and continuous variables described through means and standard deviation.

Multiple imputation by chained equations was used to replace missing values in the study sample. A total of 5 imputed data sets were generated and after applying the regression models to each, the results were pooled and adjusted to create the overall estimate, standard error, and p-value for each of the models [35].

The base relationships between physical activity and each of the three categorical geriatric-relevant health outcomes was computed using proportional odds logistic regression; linear regression was used for depressive symptoms. The regression models were adjusted for age, education, and household income, and version 1.2 of the CLSA’s sampling weights were applied [38]. The same adjusted regressions were run for female-only and male-only subsets of the study participants to allow for sex-based analysis. The addition of greenness or walkability as an interaction term to the base regression models allow for the assessment of whether there was any moderation effect on the base relationships between physical activity and the geriatric syndromes [39]. The threshold for significance was set at α = 0.05, and is reported in both 95% confidence intervals and p-values. To help compensate for the overestimation of precision that occurs when using statistical modelling on large sample sizes, the base associations were only deemed to be significant if their p-values were p <.001 instead of p <.05 [40].

## Results

Sample characteristics for the entire sample (age 73.0 ± 5.6 years), only female participants (age 73.0 ± 5.7 years), and only male participants (age 72.9 ± 5.6 years) are presented in Table 1. More than half of the participants reported having completed a ‘College/CEGEP Diploma’, ‘Bachelor’s Degree’ or ‘Graduate Degree’ as their highest level of formal education. The mean amount of weekly total physical activity hours across the whole sample was 4.0 ± 5.5 h, was right skewed, and likely contains some amount of zero inflation. This is common in measures of physical activity and did not affect the models selected for use in these analyses since physical activity is not an outcome variable for the models [41].

**Table 1:** Sample Characteristics by Self-Reported Sex (n = 16,735)

		Overall (n = 16,735)	Female (n = 8,372)	Male (n = 8,363)
Age (x̄±SD)		72.96 (5.64)	72.99 (5.71)	72.93 (5.58)
Total weekly physical activity hours (x̄±SD)		4.03 (5.48)	3.51 (4.76)	4.54 (6.08)
Depressive Symptoms (x̄±SD)		5.25 (4.43)	5.79 (4.65)	4.71 (4.12)
Physical Impairment (%)	No Impairment	12,534 (74.9)	5506 (65.8)	7028 (84.0)
	Mild	2980 (17.8)	2085 (24.9)	895 (10.7)
	Moderate	489 (2.9)	256 (3.1)	233 (2.8)
	Severe	95 (0.6)	61 (0.7)	34 (0.4)
	Total Impairment	52 (0.3)	21 (0.3)	31 (0.4)
	Missing	585 (3.5)	443 (5.3)	142 (1.7)
Severity of Daily Pain (%)	No Pain	10,416 (62.2)	4746 (56.7)	5670 (67.8)
	Mild	2062 (12.3)	1024 (12.2)	1038 (12.4)
	Moderate	3201 (19.1)	1959 (23.4)	1242 (14.9)
	Severe	693 (4.1)	437 (5.2)	256 (3.1)
	Missing	363 (2.2)	206 (2.5)	157 (1.9)
Number of Medications (%)	None	646 (3.9)	336 (4.0)	310 (3.7)
	One	879 (5.3)	483 (5.8)	396 (4.7)
	Two	990 (5.9)	536 (6.4)	454 (5.4)
	Three or More	3623 (21.6)	1755 (21.0)	1868 (22.3)
	Missing	118 (0.7)	61 (0.7)	57 (0.7)
	N/A	10,479 (69.2)	5201 (62.1)	5278 (63.1)
Education Levels (%)	Grade 8 or Less	517 (3.1)	292 (3.5)	225 (2.7)
	Grade 9 or 10	738 (4.4)	418 (5.0)	320 (3.8)
	Grade 11 to 13	411 (2.5)	223 (2.7)	188 (2.2)
	Highschool Graduate	1956 (11.7)	1115 (13.3)	841 (10.1)
	Some Post-Secondary	1316 (7.9)	682 (8.1)	634 (7.6)
	Trade Certificate/Diploma	1866 (11.2)	793 (9.5)	1073 (12.8)
	College/CEGEP Diploma	2490 (14.9)	1738 (20.8)	752 (9.0)
	University Non-Degree Certificate	789 (4.7)	439 (5.2)	350 (4.2)
	Bachelor’s Degree	3178 (19.0)	1500 (17.9)	1678 (20.1)
	Graduate Degree	3325 (19.9)	1103 (13.2)	2222 (26.6)
	Other	86 (0.5)	44 (0.5)	42 (0.5)
	Missing	63 (0.4)	25 (0.3)	38 (0.5)
Household Income (%)	Less than $20,000	1043 (6.2)	763 (9.1)	280 (3.3)
	[$20,000, $50,000)	5521 (33.0)	3263 (39.0)	2258 (27.0)
	[$50,000, $100,000)	6055 (36.2)	2560 (30.6)	3495 (41.8)
	[$100,000, $150,000)	1776 (10.6)	585 (7.0)	1191 (14.2)
	$150,000 or more	920 (5.5)	246 (2.9)	674 (8.1)
	Missing	1420 (8.5)	955 (11.4)	465 (5.6)
Walkability (%)	1 – Very Low	4694 (28.0)	2252 (26.9)	2442 (29.2)
	2	5557 (33.2)	2699 (32.2)	2858 (34.2)
	3	4580 (27.4)	2398 (28.6)	2182 (26.1)
	4/5 – High/Very High	1845 (11.0)	989 (11.8)	856 (10.2)
	Missing	59 (0.4)	34 (0.4)	25 (0.3)
Greenness (%)	1st – Lowest	4126 (24.7)	2214 (26.4)	1912 (22.9)
	2nd	4126 (24.7)	2127 (25.4)	1999 (23.9)
	3rd	4126 (24.7)	2040 (24.4)	2086 (24.9)
	4th – Highest	4125 (24.6)	1874 (22.4)	2251 (26.9)
	Missing	232 (1.4)	117 (1.4)	115 (1.4)

**Fig. 1:**
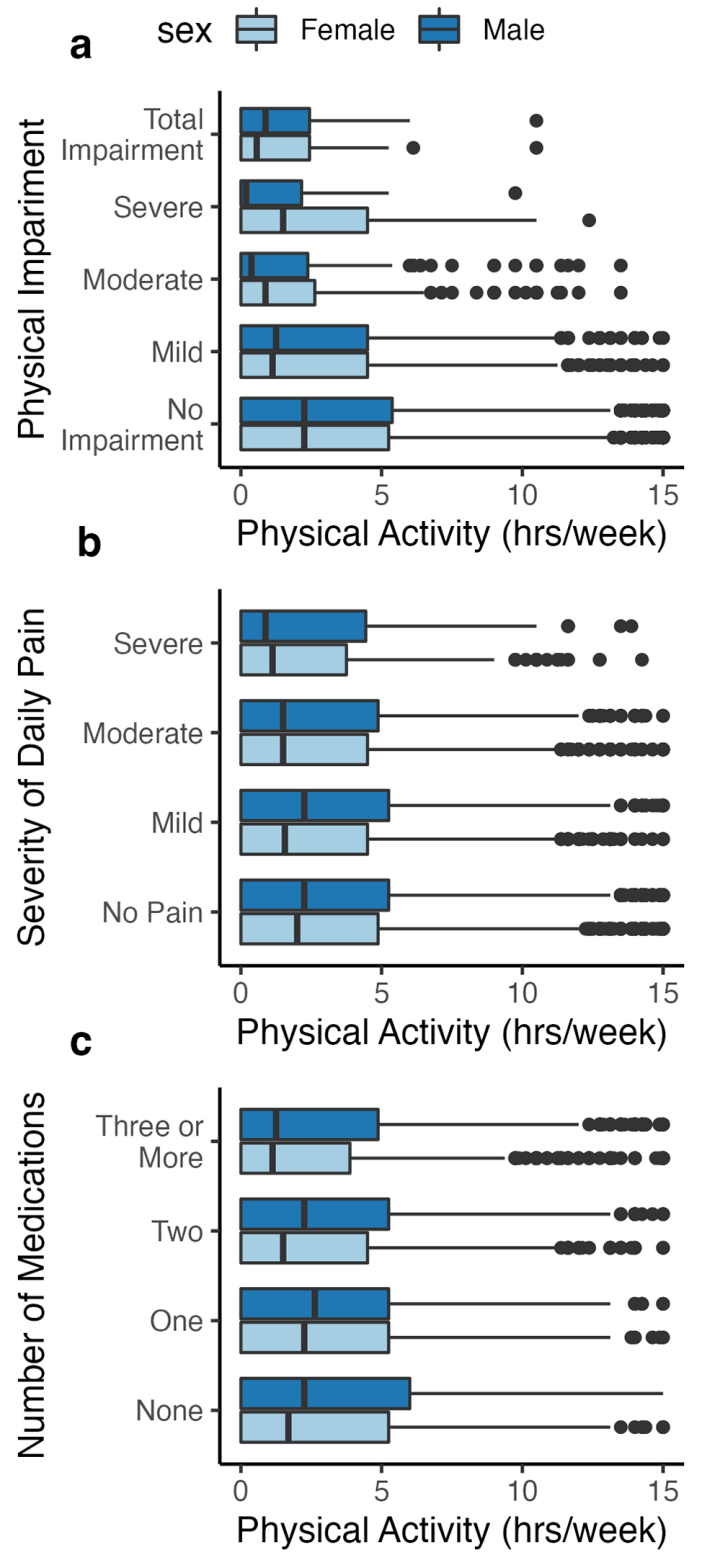
Associations of weekly total physical activity with physical impairment, severity of daily pain, and number of medications by sex Box & whisker plot of the associations between physical activity and physical impairment (a), severity of pain (b), number of medications (c) disaggregated by sex. The size of the boxplots represents the interquartile range of weekly physical activity for a given severity of the geriatric-relevant health outcome. The solid bar within the boxplot is the mean physical activity value and the dots beyond the whiskers represent outlier observations

**Fig. 2:**
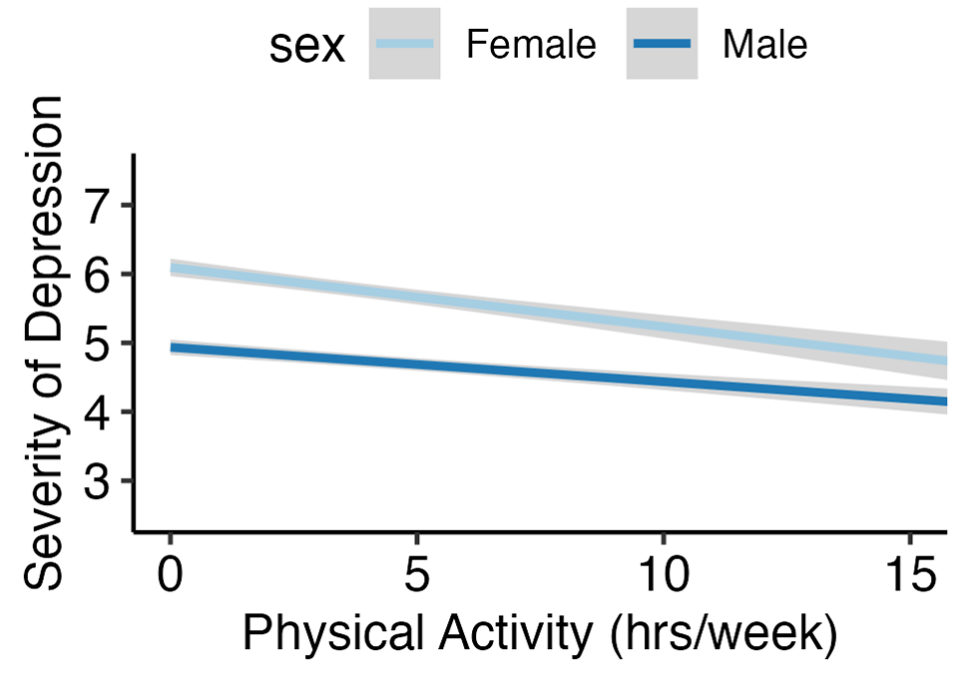
Association between weekly total physical activity and depressive symptoms by sex Line plot of association between physical activity and depressive symptoms disaggregated by sex. Shading along line represents standard error at alpha = 0.05

Associations between geriatric-relevant health outcomes and weekly physical activity are represented visually by sex in Figs. 1 and 2. Overall, lower outcome severity was associated with higher volumes of total physical activity in both male and female participants. The larger variability of weekly total physical activity among male participants is seen in the larger interquartile range of nearly all the boxplots in Fig. 1. For example, Fig. 1(b) indicates that the median weekly total of physical activity is similar in males and females who report experiencing moderate and severe pain, but the median for males experiencing mild or no daily pain is approximately 1 h per week higher than females reporting the same daily pain intensity. The associations between volume of weekly total physical activity and daily pain severity, and amount of weekly total physical activity and number of medications were not significant when only male participants were analyzed but remained significant when only female participants were analyzed. Females in our sample who were more active also had lower depressive symptom scores [which can be seen by the steeper downward slope] than males who were active the same number of hours per week (Fig. 2).

Odds ratios with 95% confidence intervals describing the base associations between each of the four measured geriatric-relevant health outcomes and weekly hours of physical activity are presented in Table 2. None of the proportional odds regression models showed evidence of separation or quasi-complete separation, and the base associations in all were significant with p-values of < 0.001. For example, for every additional 1 h of weekly physical activity a participant engaged in, the likelihood of them having experienced more severe physical impairment was approximately 5.1% lower (OR: 0.95; 95% CI: 0.94–0.96).

**Table 2:** Moderation of Greenness and Walkability on Activity and Geriatric-Relevant Health Outcomes among Older Canadians

		OR & 95% CI per Weekly Hour of TPA		Greenness (NDVI)			Walkability (Can-ALE)		
	Outcome		Type of Regression	*Moderates Base OR*	Moderation Coefficient	*p-value*	*Moderates Base OR*	Moderation Coefficient	*p-value*
Total Sample (n = 16,735)	Physical Impairment	0.95 (0.94, 0.96)	POLR	Yes	0.022	0.04	No	N/A	> 0.19
	Severity of Daily Pain	0.98 (0.98, 0.99)	POLR	Yes	0.019	< 0.01	No	N/A	> 0.34
	Number of Medications	0.98 (0.97, 0.99)	POLR	No	N/A	> 0.42	No	N/A	> 0.56
	Depressive Symptoms	0.95 (0.94, 0.96)	LR	Yes	0.032	0.01	No	N/A	> 0.19
Females (n = 8,372)	Physical Impairment	0.96 (0.94, 0.97)	POLR	No	N/A	> 0.12	No	N/A	> 0.23
	Severity of Daily Pain	0.98 (0.97, 0.99)	POLR	No	N/A	> 0.22	No	N/A	> 0.26
	Number of Medications	0.97 (0.95, 0.98)	POLR	No	N/A	> 0.19	No	N/A	> 0.33
	Depressive Symptoms	0.93 (0.91, 0.95)	LR	Yes	0.050	0.02	No	N/A	> 0.47
Males (n = 8,363)	Physical Impairment	0.95 (0.93, 0.96)	POLR	No	N/A	> 0.12	No	N/A	> 0.21
	Severity of Daily Pain	0.99 (0.98, 1.00)^a^	POLR	Yes	0.021	0.03	No	N/A	> 0.77
	Number of Medications	0.99 (0.98, 1.00)^a^	POLR	No	N/A	> 0.14	No	N/A	> 0.31
	Depressive Symptoms	0.97 (0.96, 0.98)	LR	Yes	0.029	0.05	No	N/A	> 0.39

*Note.* Abbreviations: POLR, proportional odds logistic regression; LR, linear regression; TPA: total physical activity; NDVI: Normalized Difference Vegetative Index; Can-ALE: Canadian Active Living Environments

^a^ OR p-value is > 0.001

The moderation effects of greenness and walkability, assessed separately, are also in Table 2. The moderation effects reported represent the additional change in the likelihood of having a geriatric-relevant health outcome that is attributable to the combination of greenness and physical activity or walkability and physical activity after the individual effects of those variables have been accounted for. Overall, our analyses found that greenness has a moderation effect on the base relationships between weekly physical activity and physical impairment, severity of daily pain, and depressive symptoms, but not on the number of medications taken. For example, the base relationship between physical activity and physical impairment was moderated by greenness with a moderation coefficient of 0.022 ($p=.04$) indicating that after adjusting for the participant’s age, education, and household income, participants who lived in areas with a higher level of greenness and physical activity also had a lower likelihood of worse physical impairment than could be explained by models that assume physical activity and greenness have independent associations with physical impairment (e.g. the same model without the interaction term of greenness and physical activity). There were no significant moderation effects found between walkability and any of the base relationships.

The moderation effect of greenness on the base associations between the four geriatric outcomes of this study and physical activity differed by sex. The moderation effect of greenness on physical activity and physical impairment seen in the entire sample ($coef=0.022, p= .04$) was no longer significant when assessing only female participants ($p>.12$) or only male participants ($p>.12$). Additionally, the moderation of physical activity and severity of daily pain remained significant in male participants ($coef= 0.021, p=.03$) but not among female participants ($p>.22$). The moderation of the association between physical activity and depressive symptoms by greenness remains significant in both females and males when assessed separately, and the moderation effect experienced by females ($coef= 0.050, p=.02$) appears to be stronger than in males ($coef=0.029, p=.05$).

## Discussion

The aims of this study were to assess the association between physical activity and geriatric-relevant health outcomes as well as the additive moderation effects of neighbourhood characteristics on this association. We found that physical impairment, severity of daily pain, number of prescription medications, and severity of depressive symptoms were all associated with total physical activity. The most novel finding of the current work is that neighbourhood greenness may have an additive moderation effect on the association between physical activity with physical impairment, pain severity, and depressive symptoms. Sex-based analysis further revealed that the additive moderation effects of greenness on physical impairment and pain severity differs between older females and males. Walkability did not moderate the relationship between physical activity and geriatric-relevant health outcomes. Our findings are the first to look at the impact of neighbourhood characteristics on the relationship between physical activity and geriatric-relevant health outcomes among older adults and have implications for the use of greenness in future research and interventions aimed at community-dwelling older adults.

The finding that neighbourhood greenness had a moderation effect on the associations between the physical activity levels of older adults and the likelihood of more severe physical impairment, daily pain, and depressive symptoms suggests that older adults who live in neighbourhoods with higher greenness have lower risk of poor geriatric-relevant health outcomes per hour of total physical activity than could be explained by summing the effects of greenness and physical activity with geriatric-relevant health outcomes separately. The mechanisms for this additive moderation in the association between greenness and physical activity are unclear; however, research on depression suggests that there may be an overlap in causal pathways such as the impact of greenness and physical activity on cortisol levels or telomere length [42–46]. It is possible that when physical activity is performed in an environment with denser greenness that these pathways are further activated. It is also possible that when physical activity is performed in environments with denser greenness that behaviour is impacted. For example, research interventions comparing indoor and outdoor exercise interventions have reported higher exercise intensity,  better mood, and improved social factors for participants in the outdoor exercise interventions [47, 48].

It is important to note that these associations are likely multi-directional. Although our exposure variables and outcome variables were three years apart, and we removed individuals from the dataset who had moved in the past three years, it is possible that geriatric-relevant health outcomes are leading to changes in physical activity levels. For example, older adults who are in more pain may be less physically active [14]. Future research with a longer follow-up period is needed to better understand temporality. Further, the lack of additive moderation effect observed for medication use suggests that we may have overlooked a confounding variable or that other mechanisms are at play. More rigorous experimental research is required to better understand whether the mechanisms proposed here meaningfully affect the development or severity of geriatric-relevant outcomes. A better understanding of the mechanisms that lead to the moderation effects seen in this study could be further translated into public health and municipal planning programs. For example, if it is established that the additional benefit to the effect of physical activity for older adults living in greener neighbourhoods is due to an increase in the amount of physical activity performed outdoors, then activity coordinators may offer more physical activity programming outdoors, or urban planners may design age-friendly communities to encourage active transportation to local markets and destinations.

Another factor that may alter the mechanistic links of the greenness moderation effect may be sex. We found that the moderation effect on physical impairment was no longer significant when we disaggregated the sample by sex. We also found that the effect on severity of pain was only significant among older males in our sample, and that the protective effects on depressive symptoms was stronger among older females. These findings indicate that biological sex influences the moderation effect of greenness, however, the inconsistencies suggest that perhaps there are y also gender differences that biological sex alone is unable to capture. For example, the socialized norms of expected behaviours among genders in the reporting of pain or depressive symptoms may have played a part the in differences seen in our findings [49]. Future research employing a sex and gender-based analysis approach is needed to further explore this; the CLSA does not contain data on participant gender.

There were no moderation effects of walkability even when education and household income were controlled for. This lack of effect is consistent with previous findings from our work that suggest the Can-ALE might not be reflective of the walkability experience of older adults [23, 50]. As pointed out by Clarke et al., the Canadian weather, particularly the snow and rain, has a significant effect on neighbourhood walkability, regardless of the intensity or density of the neighbourhood [51]. These environmental barriers and municipal approaches have been shown to significantly impact walkability among older Canadians [52]. A tool assessing the perceived walkability of a neighbourhood that addresses weather and infrastructure related barriers should be developed for future research in this area.

As expected, we found that total physical activity was associated with physical impairment, chronic pain, number of prescription medications, and depressive symptoms in older Canadians. These base associations add to the body of evidence that suggest a strong association between physical activity and geriatric-relevant outcomes, with this analysis in particular adding the context of community-dwelling older Canadians [11, 12, 14–16, 18]. When these associations were analyzed by sex, we found the associations with pain and medication use were no longer significant among males. Here again, it is possible that sex and gender-based analysis approaches could provide more insight.

### Strengths and Limitations

The strengths of our study include the large pan-Canadian sample, the longitudinal dataset, and access to measures of neighbourhood characteristics which enabled nuanced analysis of the effects that physical activity and neighbourhood characteristics have on geriatric-relevant health outcomes. Of note, although we used 3 year follow up data, this short follow-up period may not be sufficient for neighbourhood characteristics to impact health outcomes. While only one in five older Canadian adults report having moved in the past five years, there is not yet consensus of how long an older adult needs to have resided in a neighbourhood to be significantly affected by it [19, 53, 54]. Data from the next follow-up were recently released and can be used to provide further insights in future studies. Further, both the NDVI and Can-ALE index are proxy measures for neighbourhood greenness and walkability. Future research should find more direct measures of greenness and walkability or address the perceptions of neighbourhood walkability when working with older adults. Another neighbourhood characteristic that should be considered is material deprivations; while this is available in the dataset, there were issues of collinearity with the walkability measure that made it unusable. This is an important area for future consideration, as neighbourhood socioeconomic status may influence physical activity levels. Future research may also consider analysis of additional geriatric-relevant health outcomes and use device-measured physical activity rather than self-reported physical activity. Physical activity that is conducted indoors versus outdoors, and for different purposes (active transportation versus leisure) may also provide further insight into healthy aging. Lastly, the analytic sample is generally healthy, affluent, and lived in urban areas.

## Conclusions

In conclusion, physical activity is associated with a lower likelihood of worse physical impairment, pain severity, prescription medication use, and intensity of depressive symptoms. The moderation effect we observed between greenness and physical activity on physical impairment, pain severity, and depressive symptoms adds complexity to the results of prior research that found a positive association between high greenness and more physical activity [1, 12, 15]. Our results suggest that future research into physical activity or neighbourhood environmental factors and geriatric-relevant outcomes can benefit from including both physical activity and the neighbourhood characteristics in its analysis. For example, accounting for moderation effects between physical activity and neighbourhood environment could help researchers better control for variability in the results of a physical activity intervention that may not be due to the intervention itself.

## Data Availability

The data that support the findings of this study are available from the Canadian Longitudinal Study on Aging, but restrictions apply to the availability of these data, which were used under license for the current study, and so are not publicly available. Data are however available from the authors upon reasonable request and with permission of the Canadian Longitudinal Study on Aging.

## List of Abbreviations

Can-ALE: Canadian Active Living Environments
CES-D10: Center for Epidemiologic Studies Short Depression Scale
CI: Confidence Interval
CLSA: Canadian Longitudinal Study on Aging
NDVI: Normalized Difference Vegetative Index
OR: Odds Ratio
PASE: Physical Activity Scale for the Elderly
